# Supplementary material for: Comparative genomics provides new insights into the diversity, physiology, and sexuality of the only industrially exploited tremellomycete: Phaffia rhodozyma
Source: BMC Genomics. 2016 Nov 9;17:901. doi: 10.1186/s12864-016-3244-7 (PMC5103461; doi:10.1186/s12864-016-3244-7)
Supplement: Additional file 6: — List of orphan genes with links to PFAM (related to Additional file 1: Table S1). (ZIP 1428 kb) [file 12864_2016_3244_MOESM6_ESM.zip › PFAM_UNIQUE/G05204_P.html]

#                                                                            --- full sequence --- -------------- this domain -------------   hmm coord   ali coord   env coord
  
# target name        accession   tlen query name           accession   qlen   E-value  score  bias   #  of  c-Evalue  i-Evalue  score  bias  from    to  from    to  from    to  acc description of target
  
#------------------- ---------- ----- -------------------- ---------- ----- --------- ------ ----- --- --- --------- --------- ------ ----- ----- ----- ----- ----- ----- ----- ---- ---------------------
  
G05204\_P             -            363 Dsh\_C                PF12316.3    203      0.57    8.9  10.1   1   2      0.19   1.2e+02    1.4   1.3    64    83   109   132    76   146 0.68 - Segment polarity protein dishevelled (Dsh) C terminal  
G05204\_P             -            363 Dsh\_C                PF12316.3    203      0.57    8.9  10.1   2   2   0.00011     0.064   12.0   1.9    46   147   161   263   152   290 0.66 - Segment polarity protein dishevelled (Dsh) C terminal  
G05204\_P             -            363 DUF4611              PF15387.1     96      0.14   11.0   1.5   1   2       1.5   3.8e+02   -0.0   0.1    47    77   192   222   177   233 0.61 - Domain of unknown function (DUF4611)  
G05204\_P             -            363 DUF4611              PF15387.1     96      0.14   11.0   1.5   2   2    0.0019       0.5    9.2   0.1    63    83   288   308   253   321 0.67 - Domain of unknown function (DUF4611)  
G05204\_P             -            363 TFIIE-A\_C-term       PF11521.3     86       5.9    5.6   9.1   1   3      0.22   3.2e+02    0.0   0.0    15    36    11    32     7    54 0.73 - C-terminal general transcription factor TFIIE alpha  
G05204\_P             -            363 TFIIE-A\_C-term       PF11521.3     86       5.9    5.6   9.1   2   3       1.7   2.6e+03   -2.9   0.7     9    25   196   211   188   226 0.42 - C-terminal general transcription factor TFIIE alpha  
G05204\_P             -            363 TFIIE-A\_C-term       PF11521.3     86       5.9    5.6   9.1   3   3   0.00022      0.32    9.6   0.8    18    54   286   322   278   331 0.77 - C-terminal general transcription factor TFIIE alpha
